# Detection of sister-species in invasive populations of the fall armyworm *Spodoptera frugiperda* (Lepidoptera: Noctuidae) from Uganda

**DOI:** 10.1371/journal.pone.0194571

**Published:** 2018-04-03

**Authors:** Michael H. Otim, Wee Tek Tay, Thomas K. Walsh, Dalton Kanyesigye, Stella Adumo, Joseph Abongosi, Stephen Ochen, Julius Sserumaga, Simon Alibu, Grace Abalo, Godfrey Asea, Ambrose Agona

**Affiliations:** 1 National Crops Resources Research Institute, Namulonge, Kampala, Uganda; 2 Commonwealth Scientific and Industrial Research Organization, Canberra, Australia; 3 National Agricultural Research Organization, Entebbe, Uganda; University of Otago, NEW ZEALAND

## Abstract

The fall armyworm (FAW) *Spodoptera frugiperda* (J. E. Smith) is a species native to the Americas. This polyphagous lepidopteran pest was first reported in Nigeria and the Democratic Republic of São Tomé and Principe in 2016, but its presence in eastern Africa has not been confirmed via molecular characterisation. In this study, FAW specimens from western and central Uganda were identified based on the partial mtDNA COI gene sequences, with mtDNA COI haplotypes matching those identified in Nigeria and São Tomé. In this study, we sequence an additional partial mtDNA Cyt *b* gene and also the partial mtDNA COIII gene in Ugandan FAW samples. We detected identical mitochondrial DNA haplotypes for both the mtDNA Cyt *b* and COI partial genes, while combining the mtDNA COI/Cyt *b* haplotypes and mtDNA COIII haplotypes enabled a new maternal lineage in the Ugandan corn-preferred FAW samples to be identified. Our results suggested that the African incursions of *S*. *frugiperda* involved at least three maternal lineages. Recent full genome, phylogenetic and microsatellite analyses provided evidence to support *S*. *frugiperda* as likely consisted of two sympatric sister species known as the corn-preferred and rice-preferred strains. In our Ugandan FAW populations, we identified the presence of mtDNA haplotypes representative of both sister species. It is not known if both FAW sister species were originally introduced together or separately, and whether they have since spread as a single population. Further analyses of additional specimens originally collected from São Tomé, Nigeria and throughout Africa would be required to clarify this issue. Importantly, our finding showed that the genetic diversity of the African corn-preferred FAW species is higher than previously reported. This potentially contributed to the success of FAW establishment in Africa. Furthermore, with the additional maternal lineages detected, there is likely an increase in paternal lineages, thereby increasing the diversity of the African FAW population. Knowledge of the FAW genetic diversity will be needed to assess the risks of introducing Bt-resistance traits and to understand the FAW incursion pathways into the Old World and its potential onward spread. The agricultural implications of the presence of two evolutionary divergent FAW lineages (the corn and the rice lineage) in the African continent are further considered and discussed.

## Introduction

Incursions by exotic insect pests can have significant impacts on the ecological, agricultural and socioeconomic landscape of the invaded country. Often caused by anthropogenic activities such as trade and tourism (e.g., [1–6]), the characteristics of the invasive species play a key role in the establishment, economic cost and the ultimate fate of the species in its new environment. A propensity for long distance dispersal represents a significant challenge for management (e.g., [6, 7]). A well-documented example is the recent incursion of the Old World cotton bollworm *Helicoverpa armigera* in Brazil [8, 9]. Suspected to have been present in Brazil since at least 2008 [10], this lepidopteran pest has since been detected in neighbouring countries of Argentina [11], Paraguay and Uruguay [12], Puerto Rico [5], the Dominican Republic [6] and Florida in the US [13].

The fall armyworm (FAW) *Spodoptera frugiperda* (J.E. Smith) (Lepidoptera: Noctuidae) is a species endemic to the North and South American continents. The species is highly polyphagous with a host range of over 80 plant species and are highly migratory. These characteristics make the FAW a significant risk to agricultural production and food security for the rest of the world including the EU, Africa, Asia and Australasia. For example, the FAW poses a significant risk to maize crops which are the staple crop grown in large parts of eastern, central and southern Africa. The FAW can also readily develop resistances to insecticides (e.g., pyrethroids, organophosphates, carbamates; [14]) leading to difficulties in its control using conventional pesticides, especially at advanced larval developmental stages. Throughout most of the South and North Americas, management of FAW on corn and cotton has relied on transgenic plants expressing one or more insecticidal proteins derived from *Bacillus thuringiensis* (Bt). Unfortunately, the FAW has shown a capacity to develop resistance to Bt proteins such as Cry1F [14–22].

Although recognised as an agricultural pest species of significant biosecurity concern by agricultural/horticultural industries and governmental departments in various countries (e.g., [23], <http://bie.ala.org.au/species/ALA_Spodoptera_frugiperda>, the European Plant Protection Organisation (EPPO) [24]), *S*. *frugiperda* had never been reported to have successfully established populations outside its native range, despite being regularly intercepted on imported plant material. Despite this, the introduction of FAW by trade has been recognised as a significant agricultural biosecurity risk (e.g., <https://gd.eppo.int/taxon/LAPHFR>, see also [24, 25]). In Africa, FAW outbreaks were first reported in January 2016 in west and central Africa (the Democratic Republic of São Tomè and Principe and Nigeria) [26]. By April 2017, it was officially confirmed in at least 11 African countries (e.g., Benin, Nigeria, Togo, Ghana, Democratic Republic of Congo, Zambia, Zimbabwe, Kenya, Mozambique, South Africa and Swaziland [27] but not in the north-central African regions including Uganda. In Zambia, the FAW affected an estimated 130,000 hectares of maize and resulted in over USD $3 million for control costs during the early stages of its introduction <http://www.fao.org/africa/news/detail-news/en/c/469532/>. By February 2017, FAW was conservatively estimated to have affected over 290,000 hectares of cropland across four countries [28]. Suspected FAW was observed in Uganda’s first 2016 cropping season, and complaints from western, eastern, central and northern Uganda began to emerge of outbreaks of “stem borers” by the second cropping season of 2016.

The route of introduction into west Africa is speculated to have been through international trade, air travel [29], climate change [28], or natural dispersion via a weather event or prevailing winds <http://www.cimmyt.org/tackling-the-deadly-fall-armyworm-infestation-devastating-maize-in-southern-africa/>. The original incursion location(s) and subsequent movements of *S*. *frugiperda* in the African continent has been proposed to have been from a region encompassing the Eastern United States and the Caribbean [30]. Adding to the challenge of understanding the population dynamics of this invasive New World lepidopteran pest is the poor species delimitation of the *S*. *frugiperda* species complex that preferentially feed on either rice and various pasture grasses (i.e., the ‘R’ or ‘Rice’ strain), or on maize/cotton/sorghum (i.e., the ‘C’ or ‘Corn’ strain) host crops. These two strains are thought to represent potential sister *S*. *frugiperda* species [31–33]. Significant genetic [34], feeding, mating behaviour and pre- and post-zygotic reproductive isolation (e.g., see [35]) were reported in natural populations [36] and laboratory maintained cultures [34] of both genetic strains. Differences in developmental duration and development stage weights were also observed among different populations/sister species of FAW when raised on their preferred host crops [31, 37], while clear phylogenetic clustering separated the maize-preferred and rice-preferred sister species when inferred from multiple evolutionary models based on the mitochondrial mtDNA COI gene [38], mtDNA COI nucleotide distances [39] and analysis of the full genome sequence [40].

In this paper, we report, for the first time, the detection of FAW populations in Uganda using molecular markers. We provide a detailed genetic diversity study of Ugandan FAW populations sampled during the 2016 cropping seasons based on three partial mtDNA gene regions (i.e., COI, Cyt *b*, and COIII), and compare our COI haplotype patterns with both São Tomé and Nigeria populations. Furthermore, we explore the power of additional DNA markers for understanding the African incursion pathways by the FAW. We discuss the implications of two FAW sister-species in Africa and highlight considerations for future management strategies for this highly damaging lepidopteran pest species complex with a propensity for evolving resistance to insecticides.

## Materials and methods

Suspected FAW larvae were collected from maize fields in western and central Uganda from July to December 2016 (S1 Table). The larvae were either placed directly in 5 ml vials containing absolute ethanol or provided with maize leaves as feed in plastic bottles. Specimens were transported to the laboratory where they were reared on maize leaves. In order to obtain adults, pupae were placed in plastic bottles and monitored for adult emergence. All samples were stored in absolute ethanol until needed for molecular analysis.

### DNA extraction

DNA of individual suspected FAW was extracted using the chelex 100 (Bio Rad Laboratories, Inc., United States) method of [41]. A total of 53 specimens were used in molecular species identification (S1 Table). Before extraction, each sample was washed in sterile distilled water and a new and sterile surgical blade was used to cut off a leg. Excised legs were placed in individual sterile 1.5mL Eppendorf tubes and 50 μl of 10% chelex 100 solution was added followed by 10 μl (20mg/ml) of proteinase K solution (www.bioline.com). Individual samples were incubated at 56°C overnight, followed by brief vortex and heat inactivation at 100°C for 15 min. The resulting mixture was centrifuged at 15,900 relative centrifugal force (rcf) for 3 minutes and then 40μl supernatant collected into a new sterile 1.5ml Eppendorf tube and stored at -20°C.

### PCR amplification

All PCR amplification reactions were carried out using a Biometra thermo cycler (Göttingen, Germany). We used 1 μl of extracted genomic DNA as template in a 25 μl PCR reaction. Each PCR reaction of 25 μl consisted of 5 μl of 5X Green Go Taq Reaction buffer (Promega, USA), 2.5 μl of 25mM MgCl_2_ (Qiagen, Germany), 0.4μM gene-specific primer pairs (COI: LCO1490 and HCO2198; 710bp [42]; Cyt *b*: Sf-Cytb-f01: ATTAATTGATTTACCTTCCCCATCT, Sf-Cytb-r01: GTTAAAGTAGCATTATCAACGGCAA, 477bp; COIII: Sf-COIII-f01: CAATTTTAGTAACCAAAGGACTTC, Sf-COIII-r01: AAAGGAATAAYCAWACTACATCTAC, 543bp), 0.2mM dNTP’s, and 2 units of DreamTaq DNA polymerase (Thermo Fisher Scientific Inc.). Primers for amplifying the mtDNA Cyt *b* and COIII partial genes were developed specifically for this project based on published *S*. *frugiperda* complete mitogenomes (KM362176 and KU877172). We used the primer analysis software Oligo version 7.6 (Molecular Biology Insights, Inc.) for design of PCR/sequencing primers for the partial Cyt *b* and COIII mtDNA genes based on previously described criteria [43].

All samples were PCR amplified for the mtDNA COI partial gene region for DNA-assisted species identification. Samples that were identified as FAW were subsequently PCR amplified for the partial mtDNA Cyt *b* and mtDNA COIII gene regions. All PCR reactions were amplified through 35 cycles using the following parameters: initial denaturation step at 95°C for 60 seconds, followed by 35 cycles of DNA denaturation/annealing/extension steps at 60 seconds (95°C)/60 seconds (52°C)/90 seconds (72°C), and a final extension step at 72°C for 7 minutes.

### PCR amplicon purification, Sanger sequencing and sequence analysis

PCR amplicons were visualized on 2% agarose gels (UltraPure Agarose, Invitrogen). All PCR products were purified using GeneJET PCR Purification Kit (Thermo Fisher Scientific Inc.) following the manufacturer’s protocol prior to being shipped to Macrogen Europe for sequencing. We used the Pre-Gap4 and Gap4 sequence analysis programs within the Staden sequence analysis package [44] to analyse the trace files. Mitochondrial DNA haplotypes for all three partial gene regions were compared to sequences in GenBank via Blast search against the non-redundant (nr) DNA database.

### Estimates of evolutionary divergence between sequences

Randomly selected *S*. *frugiperda* sequences overlapping our 638bp partial mtDNA COI gene region were trimmed prior to estimating evolutionary divergences between the mtDNA COI sequences based on the uncorrected pair-wise nucleotide distances (*p*-distance) method (see [45]). Additionally, the partial mtDNA COI, Cyt *b*, and COIII genes representing each of the three maternal lineages identified from Uganda were concatenated to form a sequence of 1,468bp (i.e., MF197867-MF197869-MF197873, = NaCRRI-01; MF197868-MF197870-MF197871, = NaCRRI-02A; MF197868-MF197870-MF197872, = NaCRRI-02B). Corresponding partial mtDNA gene regions from previously reported complete mtDNA genomes of two *S*. *frugiperda* (KU877172, K362176) and the mitogenomes of the rice- and corn-preferred FAW that were generated as part of the FAW genome study [40] were also extracted and concatenated for pairwise intraspecific *p*-distance estimation. To enable comparison of the uncorrected *p*-distance values obtained with other known examples of closely related/sister species, we looked to the Old World cotton bollworm *H*. *armigera* and its closely related New World relative *H*. *zea* as an example of expected *p*-distance between possible sister heliothine species. We also compared the two *Helicoverpa* species with *H*. *punctigera* that is known to be a distinct and ancestral species to provide a guideline for expected between species *p*-distances. We used MEGA 7 [46] for all estimates of evolutionary divergence between sequences using the uncorrected *p*-distance method, with standard error (s.e.) estimates obtained by via 500 bootstrap replications. We included all three codon positions, and allowed pairwise deletion for missing data. Nucleotide distances considered all transitions and transversion substitutions with uniform rates among sites.

### Phylogenetic relationship between rice- and corn-preferred FAW

We used the Geneious Tree Builder program within Geneious v8.1.9 to infer the phylogenetic relationships between the two partial mtDNA COI haplotypes (MF197867 and MF197868) that we identified in our Ugandan *S*. *frugiperda* samples. To infer the phylogenetic positions of the mtDNA COI haplotypes detected in the Ugandan *S*. *frugiperda* individuals, we included 36 randomly selected ‘*S*. *frugiperda*’ mtDNA COI gene sequences obtained from GenBank (S2 Table) and included also *S*. *litura* and *S*. *exigua* as out groups. Furthermore, we included reference corn and rice strains from the complete mitogenomes assembled by Gouin et al. [40]. Sequence alignment of these 42 partial mtDNA COI gene regions was carried out in Geneious v8.1.9 using MAFFT v7.017 [47] and implementing default options (Auto algorithm, Scoring matrix: 200PAM / k = 2, Gap open penalty: 1.53, Offset value: 0.123). We also carried out a multigene phylogenetic analysis based on three partial mtDNA gene regions that represented three maternal lineages in FAW populations from Uganda. For this multi-gene analysis, we aligned our partial mtDNA COI, COIII, and Cyt *b* genes from the three putative maternal lineages (i.e., NaCRRI-01; NaCRRI-02A; NaCRRI-02B) against publicly available full mitochondrial DNA genomes of *FAW* (i.e., Rice-REF, Corn-REF; <http://bipaa.genouest.org/data/public/sfrudb/FAW_mitochondrial_genomes.fa>, [40]); KU877172, KM362176, [48]) and the related *S*. *exigua* (JX316220; [49]) and *S*. *litura* (JQ647918; [50]) (see S2 Table). For the partial mtDNA COI gene phylogenetic analysis, we implemented the Neighbor-Joining (NJ) tree building method and the Hasegawa-Kishino-Yano (HKY) [51] genetic distance model. We chose the HKY model as this allows for unequal base frequencies and distinguishes between transition and transversion rates, and is most similar to the criterion for the multi-gene evolutionary model. For the multigene phylogenetic analysis, we used the web-based PhyML 3.0 program [52] with the automatic model selection by SMS option’ [53] with selection criterion set as ‘AIC’ (Akaike Information Criterio), with 1,000 bootstrap replications to ascertain node confidence between branches. Trees were bootstrapped (sampled with replacement) for 1,000 replicates to produce a consensus tree with node support of ≥50% shown.

## Results

### FAW mtDNA partial genes characterisation

**Fig 1:**
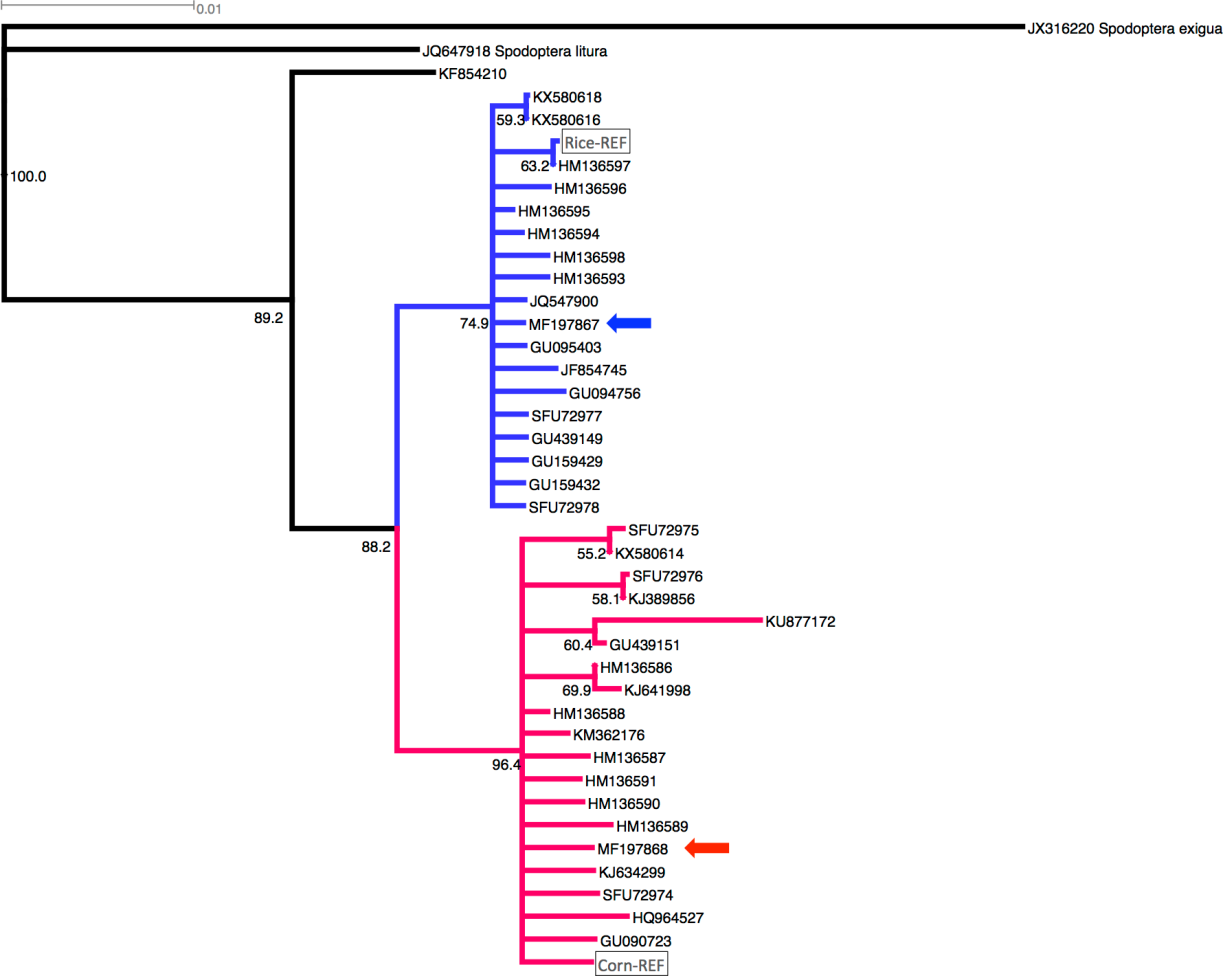
Phylogenetic analysis of *Spodoptera frugiperda* selected partial mitochondrial DNA COI (mtDNA COI) sequences obtained from publicly available DNA database, including Corn-REF and Rice-REF (see <http://bipaa.genouest.org/data/public/sfrudb/FAW_mitochondrial_genomes.fa>) haplotypes, and haplotypes identified in this study (MF197867, indicated by blue coloured arrow; MF197868, indicated by red coloured arrow). Rice-preferred and corn-preferred mtDNA COI haplotypes were confidently clustered into sister clades at 74.9% and 96.4%, respectively. The partial mtDNA COI haplotype KF854210 identified also as *S*. *frugiperda* (isolate GK424, [38] was inferred to be basal to the *S*. *frugiperda* rice and corn sister clades. The out groups species were *S*. *exigua* (JX316220) and *S*. *litura* (JQ647918).

Of the suspected 53 FAW specimens, two individuals (i.e., 61 and 79) failed to PCR amplify, one (i.e., MF197865) matched *H*. *armigera* (99%, KF661360.1), one (i.e., MF197866) matched a Noctuidae species (92%, KC172801), and 49 were identified as *S*. *frugiperda* from maize from six western (i.e., Buliisa, Kabarole, Kamwenge, Kasese, Kibale and Kiryandongo) and central (i.e., Wakiso) districts in Uganda. We detected the two previously reported *S*. *frugiperda* mtDNA COI haplotypes based on 638bp trimmed partial mtDNA COI gene (i.e., KX580616 and KX580618 matching the mtDNA haplotype MF197867 from this study; and KX580614 matching the mtDNA haplotype MG197868 from this study; see Fig 1). Two partial mtDNA Cyt *b* haplotypes (trimmed to 380bp; MF197869, MF197870) were also detected that matched the partial mtDNA COI haplotype patterns in our sampled FAW populations. However, based on the mtDNA COIII partial gene region characterised (trimmed to 450bp; trace files available upon request), we identified three maternal lineages (MF197873, MF187871, MF197872) within the 49 FAW individuals. A total of 32 single nucleotide polymorphisms (SNPs)/base changes were detected across the three partial mtDNA gene regions for COI (11 SNPs), COIII (13 SNPs) and Cyt *b* (8 SNPs), of which 28 were transition and four transversion base changes (S3 Table).

The majority of the mtDNA COI sequences retrieved from GenBank originated from the New World with the exception of both Nigerian (KX580616, KX580618) and São Tomé (KX580614) sequences representing the new invasive mtDNA COI haplotypes (see Fig 1). The New World sequences originated from the USA (including its offshore territory of Puerto Rico), Canada, Peru, and Brazil. When compared with the 638bp partial mtDNA COI sequences from Uganda, both African haplotypes matched completely to individuals sampled from the USA and Canada (S2 Table).

The partial mtDNA Cyt *b* haplotype MF197869 returned six matches that were 100% (HQ177669, HQ177667), 98% (KM362176, HQ177674, HQ177673) and 96% (KU877172) similar. The second Cyt *b* haplotype (MF197870) matched 100% to three GenBank entries (KM362176, HQ177674, HQ177673), and also three GenBank entries (i.e., KU877172, HQ177669, HQ177667) at 98% sequence homology. For both Cty *b* haplotypes with 100% GenBank entries matches, these individuals were reported to have originated from Guadeloupe (HQ177667) and French Guiana (HQ177669), as well as from Guadeloupe (HQ177674) and Peru (HQ177673) [39], and also included a *S*. *frugiperda* (Sf) cell line (KM362176, [48]). Of the three mtDNA COIII haplotypes, one (MF197871) matched 100% to a GenBank record (i.e., M22051; [54]) that originated from a Sf cell line, and to two GenBank records at 98% (KU877172) and 93% (KM362176; [48]) sequence homology. The COIII haplotype (MF19787) matched GenBank records M22051, KU877172 and KM362176 at 99%, 97% and 93% sequence homology, respectively. The third mtDNA COIII haplotype detected (MF197873) matched 95% and 97% to KU877172 and KM362176, respectively.

### Uncorrected nucleotide (*p*-) distances

**Fig 2:**
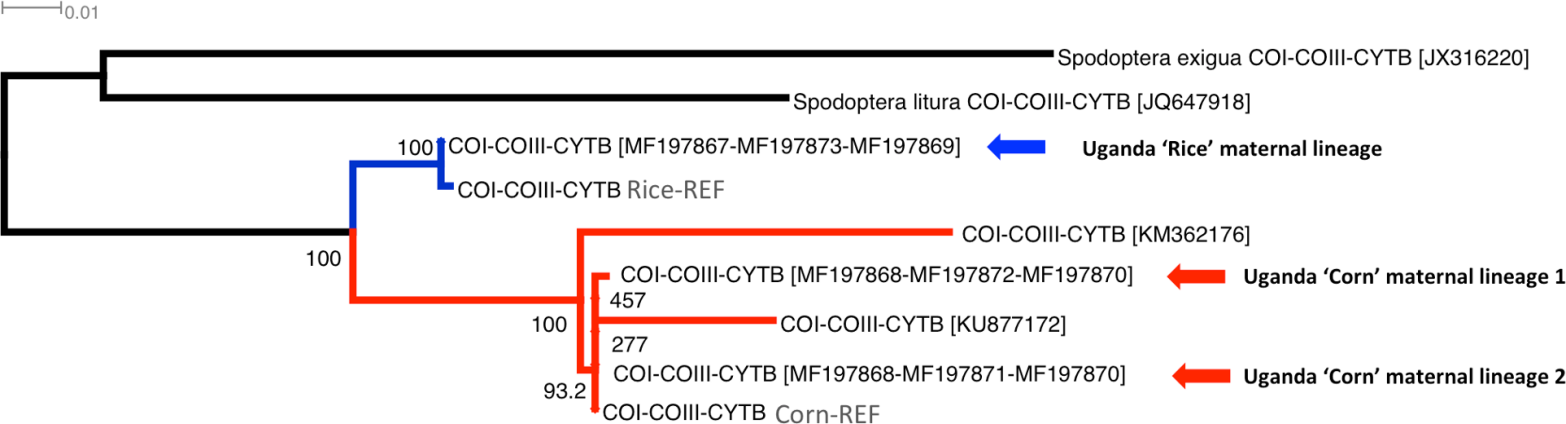
Multigene phylogeny (i.e., from concatenation of partial mitochondrial DNA (mtDNA) COI, COIII, Cyt *b* genes) of the rice-preferred and corn-preferred Ugandan *Spodoptera frugiperda* sister species. Partial mtDNA COI, COIII and Cyt *b* gene sequences were obtained from full mtDNA genomes of *Spodoptera* species (*S*. *exigua* JX316220; *S*. *litura* JQ647918; *S*. *frugiperda* KM362176; *S*. *frugiperda* KU877172, and *S*. *frugiperda* ‘Corn’ and ‘Rice’ reference mitogenomes (i.e., Corn-REF, Rice-REF, <http://bipaa.genouest.org/data/public/sfrudb/FAW_mitochondrial_genomes.fa>, see [40]) and from sequences generated through this study (MF197867, MF197873, MF197869 (i.e., Uganda ‘Rice’ maternal lineage); MF197868, MF197871, MF197870 (i.e., Uganda ‘Corn’ maternal lineage 1); MF197868, MF197872, MF197870 (i.e., Uganda ‘Corn’ maternal lineage 2)). The multigene phylogeny provided strong bootstrap support values (1,000 replications) for the existence of two closely related monophyletic *S*. *frugiperda* sister clades. The rice-preferred *S*. *frugiperda* clade is made up of two individuals (i.e., published [40]; and ‘Uganda ‘Rice’ maternal lineage’ (this study)). The corn-preferred clade has five individuals, of which two (i.e., Uganda ‘Corn’ maternal lineage 1; Uganda ‘Corn’ maternal lineage 2) are from this study. The remaining three ‘Corn’ maternal lineages are from published sequences (i.e., [40]; KM362176; KU877172). Out group species are *S*. *exigua* and *S*. *litura*.

Our estimated nucleotide distances between the 36 randomly selected mtDNA COI partial gene sequences can be broadly divided into the rice and corn groups (S2 and S4 Tables). The rice group (equivalent to ‘Rice strain’) included the haplotype (MF197867) that also matched the Nigeria partial mtDNA COI (KX580616, KX580618). Nucleotide distances for all rice group individuals ranged between 0 and 0.004 (variance range 0 and 0.003). The corn group (equivalent to ‘Maize/Corn strain’) included the haplotype (MF187868) that matched the São Tomé haplotype (KX580614). The estimated pairwise nucleotide *p*-distances for all corn group sequences also ranged between 0 and 0.04 (variance ranged between 0 and 0.003). Both rice and corn group sequences (S2 Table) have been shown [38] to be characteristic of the rice-preferred and corn-preferred sister-species of FAW, respectively. Other publicly available sequences (e.g., KM362176, KJ634299, KJ389856, HQ964527, KJ641998; S4 Table) were also found to share very close nucleotide distances with the corn group sequences (ranged between 0 to 0.005; 0–0.003 standard error (s.e.)). Nucleotide distances between rice and corn group sequences generally ranged between 0.012 and 0.022 (0.005–0.006 s.e.), and was similar to previously reported average divergence estimate of 0.0209 between both sister species [39] based on the Kimura 2-parameter nucleotide evolutionary model. Two reported *S*. *frugiperda* partial mtDNA COI sequences (i.e., KU877172, KF854210) showed much higher pairwise nucleotide sequence distances, especially between KU877172 and rice group sequences (i.e., 0.021 to 0.028, ± 0.06 s.e.), and between KF854210 and corn group sequences (0.028 to 0.032, ± 0.007 s.e.). The point estimate of 0.037 nucleotide distance between KU877172 and KF854210 (S4 Table) is similar to that reported for the closely related Noctuidae *H*. *armigera* and *H*. *zea* (i.e., *p*-distance ranged from 0.031–0.047) which represent two closely related sister species respectively, estimated to have diverged at *ca*. 1.5 million years ago (mya) [55–57] and showed no reproductive asymmetry. Multigene (i.e., concatenation of partial mtDNA COI+COIII+Cyt *b* genes) pairwise *p*-distance estimates of rice- and corn-preferred *S*. *frugiperda* individuals as presented in Fig 2 are also provided in Table 1.

**Table 1:** Estimates of evolutionary divergence (based on *p*-distance method) between concatenated partial mtDNA COI, COIII and Cyt *b* sequences of rice-preferred (COI-COIII-CYTB Rice-REF; Uganda 'Rice' maternal lineage) and corn-preferred (KM362176; KU877172; COI-COIII-CYTB Corn-REF; Uganda 'Corn' maternal lineage 1, Uganda 'Corn' maternal lineage 2) *S*. *frugiperda* individuals.

		1	2	3	4	5	6	7
**1**	COI-COIII-CYTB Rice-REF	-	0.0007	0.0053	0.0047	0.0038	0.0038	0.0039
**2**	Uganda 'Rice' maternal lineage	0.0007	-	0.0052	0.0047	0.0038	0.0038	0.0039
**3**	COI-COIII-CYTB [KM362176]	0.0429	0.0416	-	0.0047	0.0038	0.0038	0.0038
**4**	COI-COIII-CYTB [KU877172]	0.0335	0.0329	0.0356	-	0.0029	0.0029	0.003
**5**	COI-COIII-CYTB Corn-REF	0.0218	0.0211	0.0238	0.0116	-	0	0.0007
**6**	Uganda 'Corn' maternal lineage 1	0.0218	0.0211	0.0232	0.0116	0	-	0.0007
**7**	Uganda 'Corn' maternal lineage 2	0.0225	0.0218	0.0239	0.0123	0.0007	0.0007	-

*S*. *frugiperda* complete mitochondrial DNA genomes (KU877172 and KM362176) were obtained from GenBank and from published data (COI-COIII-CYTB Rice-REF, COI-COIII-CYTB Corn-REF; [40]). Concatenated partial mtDNA sequences are: MF197867-MF197869-MF197873 (representing Uganda ‘Rice’ maternal lineage’); MF197868-MF197870-MF197871 (representing Uganda ‘Corn’ maternal lineage 1), and MF197868-MF197870-MF197872 (representing Uganda ‘Corn’ maternal lineage 2). Standard error (s.e.) estimates are shown above the diagonal and were obtained from 500 bootstrap replications. All codon positions from a total of 1,468 base pairs were included in the analysis.

The two mtDNA COI haplotypes (MF197867 and MF197868) confidently grouped with the rice and corn strains/sister species, at 74.9% and 96.4%, respectively (Fig 1). These two FAW ‘strains’ were also confidently assigned as sister clades with an 88.2% bootstrap node value. Whole genome SNP phylogenies also support the existence of these two clades [40], estimated to have diverged at approximately 2 mya and with partial mating compatibility. Phylogenetic analysis based on concatenation of three partial mtDNA genes from our Ugandan FAW samples and publicly available *S*. *frugiperda*, *S*. *litura* and *S*. *exigua* mitochondrial DNA genomes clearly supported the presence of three Uganda FAW lineages (Uganda ‘Rice’ maternal lineage, Uganda ‘Corn’ maternal lineages 1 and 2; Fig 2), with the two Ugandan ‘Corn’ maternal lineages confidently clustered at 93.2%. The multigene phylogenetic analysis also returned 100% node confidence value between the five Corn lineages, and also 100% node confidence value between the Corn FAW and Rice FAW mtDNA haplotypes (Fig 2).

Our estimates of uncorrected *p*-distances obtained from the concatenation of three partial mtDNA genes between the corn- and rice- preferred FAW haplotypes ranged between 0.0211 ± 0.0038 s.e. and 0.0429 ± 0.0053 s.e. (Table 1; average = 0.281 ± 0.004 s.e.), and were similar to the estimate obtained between the two closely related noctuid moth species of *H*. *armigera* and *H*. *zea* (Table 2; *H*. *armigera* and *H*. *zea* = 0.0293 ± 0.0043 s.e.), with the *p*-distance values overlapping each other when standard error estimates were taken into consideration. The mitogenome KM362176, which occupied the basal position to the ‘corn’ clade (Fig 2) was the most diverge with an average *p*-distance of 0.0266 ± 0.004 s.e. to the other four ‘corn’ FAW maternal lineages (Table 1). Excluding this divergent KM362176 mitogenome-derived partial sequences, the corn-clade FAW concatenated partial mtDNA genes (KU877172, Corn-REF; [40]; Uganda ‘corn’ maternal lineages 1 and 2) shared an average of 0.0062 ± 0.0017 s.e. nucleotide *p*-distance), with the low nucleotide distance as expected at the intra-species level (e.g., 0.0026 ± 0.0018 s.e.; see e.g., [55]). These *p*-distance estimates supported the suggested presence of potential sister species complexes [31] within the currently recognised FAW species, and lends support for two sister-species of FAW in Africa and in Uganda.

**Table 2:** Estimates of evolutionary divergence (*p*-distance method) between concatenated partial mtDNA COI, COIII and Cyt *b* sequences of the two closely related *Helicoverpa* sister species of *H*. *armigera* (GU188273) and *H*. *zea* (KJ930516) and between the two distinct *Helicoverpa* species, i.e., *H*. *punctigera* (KF977797) and *H*. *armigera* (GU188273).

	*H*. *zea*_KJ930516	*H*. *armigera* GU188273	*H*. *punctigera*_KF977797
*H*. *zea*_KJ930516		0.0043	0.0056
*H*. *armigera* GU188273	0.0293		0.0062
*H*. *punctigera*_KF977797	0.0668	0.0674	

Standard error (s.e.) estimates are shown above the diagonal and were obtained from 500 bootstrap replications. All codon positions (1,468 base pairs) were included in the analysis.

## Discussion

In this investigation, we report on the detection of *S*. *frugiperda* in Uganda using the mtDNA COI partial gene, where both mtDNA COI haplotypes identified in Nigeria, São Tomé [26], Ghana [58], and Togo [30] were found in Uganda. Importantly, by also characterising two additional partial mtDNA gene regions (i.e., COIII and Cyt *b*), we demonstrated that seven Ugandan *S*. *frugiperda* individuals (i.e., individuals 4, 109, 112, 10A, 1A, 3A, and 5A; S1 Table) that matched the São Tomé mtDNA COI haplotype in fact consisted of two closely related maternal lineages (Fig 2), suggesting that incursions of *S*. *frugiperda* into the African continent involved at least three maternal lineages. Despite the identification of a new maternal lineage, the number of mitochondrial haplotypes remained low when compared with recent findings of *H*. *armigera* in South America (e.g., [6, 9, 12, 59–61]). The use of multiple mtDNA and nuclear DNA genes as markers can refine the identification of origins and/or pathways of introduction (e.g., [6, 30, 62, 63]; see also review by [64]). Our study showed that the inclusion of the mtDNA COIII partial gene regions increased the detection power of maternal lineages and should be included in future studies on the spread of FAW populations across the Old World.

The timing and initial incursion(s) of *S*. *frugiperda* into the African continent have yet to be determined, however, our finding of low number of matrilines despite increased mtDNA gene markers suggests a limited incursion by FAW. A paucity of samples analysed for the mtDNA COI gene region [26], as well as limited molecular characterisation of FAW populations in the New World makes ascertaining possible New World population origins and African incursion sites difficult [30]). However, we postulate two hypotheses relating to the introduction of FAW into Africa: (i) that the introduction of FAW into Africa involved several different matrilines potentially representing multiple independent introductions from the New World; and (ii) that *S*. *frugiperda* populations from São Tomé and Nigeria are potentially serving as source populations for the species’ spread across mainland Africa.

For hypothesis (i), we showed the presence of both highly related mtDNA haplotypes (i.e., MF197870 and MF197872, nucleotide similarity >99.9%) as well as highly diverged mtDNA haplotype patterns (i.e., MF197867 vs. MF197868 (see S2 Table); MF197869 vs. MF197870; MF197873 vs. MF197871; MF197873 vs. MF197872) in Uganda. Taking into consideration findings from this and previous studies (e.g., [26, 34, 38]) suggests that these populations may represent independent introductions especially if they have separate African incursion sites, while individuals possessing the partial mtDNA COIII haplotypes MF197871 and MF197872 represented two evolutionary closely related maternal lineages (Table 1; Fig 2) that potentially shared geographically proximate New World origins and were potentially introduced to Africa as a single event.

Dumas et al. [38] using multiple molecular species delimiting methods, including the Generalized Mixed Yule Coalescent approach [65, 66] and the Poisson-Tree-Processes [67], found statistical support for the presence of two sister *S*. *frugiperda* species, broadly grouped into either the rice-preferred (MF197867, this study) or the corn-preferred (MF197868, this study) host groups (S2 and S4 Tables; Figs 1 and 2). These two host-plant related sister *S*. *frugiperda* species shared identical mtDNA COI partial sequences as those detected in Nigeria and São Tomé, and were both present in our Ugandan samples collected from maize host. Identifying closely related lepidopteran species is possible using genome-wide SNPs [68] and has been applied, via whole genome sequencing, to closely related *H*. *armigera* and *H*. *zea* [57] and to both maize- and rice-preferred *S*. *frugiperda* [40]. The availability of *S*. *frugiperda* genomes [40] also paves the way for future studies of gene flow patterns and species diversity of this invasive pest complex in Africa, while also offering opportunities to identify signatures of selection pressure (i.e., adaptation to novel climatic conditions, exposure to insecticidal chemistries). The presence of both putative *S*. *frugiperda* sister species in Africa may add a new dimension to the management challenge of this pest in the Old World. Rice is the major carbohydrate in many Asian countries, and the spread of FAW populations with greater developmental fitness when fed on rice may increase the agricultural biosecurity threat for these regions. While each of the sister species may have better developmental performance when feeding on their preferred host crops [37], hybrid fitness studies are inconclusive (e.g., [33, 34], but see also e.g., [69–71]). Whether such hybridisation can contribute to better host or climate adaptation in the invasive range will require investigation. It is interesting to note that individuals reported from Nigeria [26] and our Ugandan populations, were collected from maize host crop but possessed the mtDNA COI haplotype matching their rice-preferred sister species (S2 Table; see also [38]) and we do not yet know the degree to which the genome of the African *S*. *frugiperda* correlates with the mitochondrial haplotype.

In *S*. *frugiperda*, with the inclusion of the mtDNA COIII gene region, we were able to demonstrate the presence of a third maternal lineage in Africa (Fig 2). The relatively low numbers of matrilines could be related to the biology of the species, where individual egg masses, each containing between 100–300 eggs are laid in tight clusters, and that just a few egg clusters on a small number of plants would produce enough individuals to enable successful establishment of populations in a new environment. It is possible that a small number of *S*. *frugiperda* individuals representing limited maternal lineages were transported across the Atlantic, potentially by factors such as wind patterns across oceans <http://www.cimmyt.org/tackling-the-deadly-fall-armyworm-infestation-devastating-maize-in-southern-africa/>, (but see [72] for projected movements of FAW and mtDNA COI haplotype frequency distributions across the Caribbean, North and South Americas), or through anthropogenic activities (e.g., by travellers, on agricultural commodities). Long distance dispersals in lepidopteran species (e.g., *Danaus plexippus* [73, 74]; *H*. *armigera* [75–77]; *S*. *exigua* [78]) including the FAW [72] are known, and was hypothesised to enable the establishment of the *H*. *zea* in the New World (e.g., [55]), while Tay et al. [6] demonstrated the potential role of global trade in increasing the risk of introducing invasive species (i.e., *H*. *armigera* in the South American continent).

*S*. *frugiperda* has been intercepted in countries outside the New World at least since pre-1984 [25]. Recent Europhyt Interceptions Annual Reports (i.e., the plant health interception, notification and rapid alert system for the EU member states and Switzerland, which is managed by the EU Commission) have data of FAW interceptions from New World countries. For example, six interceptions of FAW in 2014 [79] on *Capsicum frutescens*, *Capsicum* sp. and *Solanum melongena* plant hosts from Dominican Republic (n = 3), Suriname (n = 2) and Mexico (n = 1). In 2015 [80], nine interceptions of *S*. *frugiperda* from Suriname were made from plant hosts such as *C*. *frutescens*, *Capsicum* sp., *Momordica charantia*, *Momordica* sp., and *S*. *melongena*. A total of 15 FAW interceptions in 2016 [81] were reported (Equador (n = 1), Suriname (n = 14)) from *Eryngium* sp., *C*. *frutescens*, *Capsicum* sp., *M*. *charantia*, *S*. *macrocarpon*, *S*. *melongena*, *Solanum* sp., and *Vigna unguiculata* plant hosts. A detailed review of such interception data and trade partners can help aid biosecurity preparedness in countries with climatic conditions suitable for establishment of FAW populations.

Georgen et al. [26] surveyed a limited number of individuals (São Tomé, n = 3; Nigeria, n = 5) and detected unique haplotypes in each country. Knowing whether both mtDNA COI haplotypes were also present in São Tomé and/or Nigeria, and with the aid of nuclear DNA markers to infer gene flow patterns (given the low genetic diversity detected by multiple mtDNA gene markers), would be necessary to infer the origins of new populations in other mainland African countries post detection of the FAW in Nigeria (i.e., hypothesis (ii)). For example, if Nigerian populations truly represented a single maternal lineage, it would suggest the Ugandan haplotype MF197868 either represented: (a) novel New World introduction events, or (b) that its arrival in Uganda involved populations that originated from São Tomé, either as direct introduction from São Tomé, or more likely as secondary introduction from the species’ east-ward spread via neighbouring African countries. Our findings especially on the FAW Corn maternal lineage 2, do not currently rule out the direct introduction of *S*. *frugiperda* to Uganda.

The potential origin(s) as well as the potential presence of sister-species of *S*. *frugiperda* could have implications for its management. Collectively as a single pest species complex, *S*. *frugiperda* has developed resistance to a number of different control measures in its native range, but the resistance status as separate sister species (i.e., as suggested by the recent phylogenetic study [38]; see also [58]) is not known. Before the era of genetically modified crops expressing insecticidal proteins derived from Bt, this species was controlled with synthetic pesticides until the development of resistance involving different modes of action [14, 15, 18, 19, 82–85]. A number of studies have been able to select for resistance in the laboratory suggesting that *S*. *frugiperda* readily develops resistance when pressured and the first report of resistance was to carbaryl, a carbamate insecticide [86]. Further studies have reported resistance in field populations to pyrethroids (Florida and Brazil) and organophosphates (Florida) [14, 87]. More recently, there have been concerns over the ability of *S*. *frugiperda* to tolerate Bt toxins, particularly to Cry1F-incorporated transgenic plants ([16, 21, 22], see also [88]). Nagoshi et al. [30] showed the absence of known Cry1F resistance alleles commonly found in Puerto Rico in the Togo populations. MtDNA COI and Tpi alleles commonly found in the Eastern United States and the Caribbean were found in Togo populations rather than those from the western USA and South America. Homing in on the pathway(s) of introduction could assist with phytosanitary, agricultural biosecurity policy development, and insecticide resistance management strategies [6, 89] in the pest’s new invasive range. This knowledge will also enable better understanding of pathways involved in the spread of FAW across central, southern and eastern African nations, and potentially their eventual spread to the other regions with suitable climatic conditions (e.g., Asia, Southern Europe and Australasia).

The implications of *S*. *frugiperda* for Africa could be profound, not only with the economic losses associated with direct feeding damage and control costs, but also through high insecticide residues and potential trade restrictions from as yet unaffected trading partner countries. Many countries and regions list *S*. *frugiperda* as a category 1 quarantine pest and it has the potential to spread throughout the topics and sub tropics. For such a polyphagous lepidopteran pest species with a significant migratory potential, as well as the availability of suitable habitats across the Indian sub-continent, Asia, South East Asia and the Pacific, there would seem to be limited natural barriers to prevent the rapid spread of FAW across the Old World, and potentially reaching Australasia. The existence of multiple maternal lineages in the corn-preferred *S*. *frugiperda* (and potentially also in the rice-preferred *S*. *frugiperda* depending on genes surveyed) is alarming as the implications on agricultural biosecurity and evolution of resistance especially to the Bt proteins such as Cry1Ab currently being tested in Uganda for stalk borer control may be significant. Increasing genetic diversity as contributions by both male and female lineages of *S*. *frugiperda* will increase the probability of introducing resistance alleles to current populations. This is especially important given that both male and female *S*. *frugiperda* demonstrated the ability to mate multiply, while multiply-mated *S*. *frugiperda* females showed random sperm usage [90]. High genetic diversity in invasive species is likely an important factor that contributes to the success of incipient population establishment and adaptation ability in novel environments and ecosystems. Depending on whether multiple maternal lineages represented concurrent or separate incursion events of FAW into the Africa continent, detection of multiple maternal lineages will also have implications on agricultural biosecurity relating to a country’s (or continent’s) ability to prevent potential on-going introduction of invasive pests. With the increased genetic diversity detected in this study, adequate and careful planning for sampling of FAW populations across the African continent will be needed to better ascertain the likely presence of the Cry1F resistance alleles known to be present in the New World.

*S*. *frugiperda*’s invasive threat from the African continent across the Europe, the Middle East, Asia and the Australasia will need to be assessed (i.e., [91]) to gain a better understanding of its overall on-going invasion threat. Effective management strategies to control populations, as well as improved biosecurity measures relating to early detection and quarantine of commodity movements may be required in unaffected countries (although this may be too late for many of the African nations) to mitigate the two fall armyworm sister-species’ anticipated march across the Old World.

## Supporting information

S1 Table *Spodoptera frugiperda* sample ID, sampling dates, sampling districts, location coordinates and life stages used in this study. All samples were collected from maize host plant. All samples were collected as larvae except individuals 11A, 12A, 14A, 15A, and 18A which were collected as adult moths. Western districts are: Buliisa, Kabarole, Kamwenge, Kasese, Kibale, Kiryandongo and central district is Wakiso.
(DOCX)

S2 Table Publicly available partial mtDNA COI gene sequences as compared to the two Ugandan *Spodoptera frugiperda* mtDNA COI haplotypes (in yellow highlight). All polymorphisms are as compared to the sequence with the GenBank accession number MF197867. Nucleotide positions are based on the MF197867 haplotype. Previously detected African haplotypes from Nigeria (KX580616, KX580618) and from São Tomé (KX580614) are highlighted in orange. Where available information on countries of collection, years and host crops had been provided. These are listed also in this supplementary table. Dates of collection, if unavailable, we used the date where the haplotypes were reported in GenBank. Uncertainty in collection/country/host crop information is indicated by '?'. Missing bases are indicated by 'N', bases identical to the consensus sequence are indicated by '.' Rice-REF and Corn-REF were aligned partial mtDNA COI gene regions from the rice and corn reference strain complete mitogenomes [40].
(XLSX)

S3 Table Majority-rule consensus nucleotide polymorphisms detected in Ugandan *Spodoptera frugiperda* specimens at three partial mitochondrial DNA (mtDNA) gene regions. A total of 11, 13 and eight single nucleotide polymorphisms (SNPs) were detected in COI, COIII and Cyt *b* genes, respectively. Note that nucleotide position 243 for the COIII gene differentiated the 'São Tomé-like mtDNA COI haplotype' into two separate maternal lineages (i.e., samples 4, 1A, 3A, 5A; and samples 109, 112, 10A).
(XLSX)

S4 Table Estimates of uncorrected nucleotide distances (p-dist) between *Spodoptera frugiperda* 36 partial mtDNA COI sequences as obtained from GenBank (accessed 20-May-2017). These 36 randomly selected mtDNA COI partial gene sequences can be broadly divided into either that of 'rice-preferred' (samples 1–18) or 'corn-preferred' (samples 19–37) sister-species of FAW (see also Fig 1). GenBank accession numbers of mtDNA COI haplotypes identified from this study are in yellow colour. GenBank accession numbers or mtDNA COI haplotypes from Nigeria (KX580616, KX580618) and São Tomé (KX580614) are highlighted in orange colour.
(XLS)
