# Supplementary material for: Detection of sister-species in invasive populations of the fall armyworm Spodoptera frugiperda (Lepidoptera: Noctuidae) from Uganda
Source: PLoS One. 2018 Apr 3;13(4):e0194571. doi: 10.1371/journal.pone.0194571 (PMC5882101; doi:10.1371/journal.pone.0194571)
Supplement: S1 Table — All samples were collected from maize host plant. All samples were collected as larvae except individuals 11A, 12A, 14A, 15A, and 18A which were collected as adult moths. Western districts are: Buliisa, Kabarole, Kamwenge, Kasese, Kibale, Kiryandongo and central district is Wakiso. (DOCX) [file pone.0194571.s001.docx]

**S1 Table:** *Spodoptera frugiperda* sample ID, sampling dates, sampling districts, location coordinates and life stages used in this study. All samples were collected from maize host plant.

| **Samples ID** | **Sample collection date** | **Sample sites** | **Latitude** | **Longitude** |
| --- | --- | --- | --- | --- |
| 100 | 27/11/2016 | Buliisa | 2.150136 | 31.48347 |
| 103 | 27/11/2016 | Buliisa | 2.183466 | 31.51674 |
| 105 | 27/11/2016 | Buliisa | 2.150136 | 31.48347 |
| 108 | 28/11/2016 | Buliisa | 1.700162 | 31.36686 |
| 109 | 27/11/2016 | Buliisa | 2.133366 | 31.50019 |
| 112 | 1/12/2016 | Kabarole | 0.700025 | 30.33353 |
| 21 | 28/11/2016 | Kamwenge | 0.366689 | 30.50017 |
| 23 | 28/11/2016 | Kamwenge | 0.316853 | 30.50006 |
| 24 | 28/11/2016 | Kamwenge | 0.316853 | 30.50006 |
| 25 | 28/11/2016 | Kamwenge | 0.350151 | 30.50019 |
| 29 | 28/11/2016 | Kamwenge | 0.316693 | 30.43357 |
| 32 | 29/11/2016 | Kamwenge | 0.316693 | 30.43357 |
| 35 | 28/11/2016 | Kamwenge | 0.366816 | 30.4836 |
| 65 | 29/11/2016 | Kamwenge | 0.316853 | 30.50006 |
| 42 | 2/12/2016 | Kasese | 0.000167 | 29.91683 |
| 43 | 2/12/2016 | Kasese | 0.000167 | 29.91683 |
| 44 | 2/12/2016 | Kasese | 0.233461 | 30.11668 |
| 45 | 2/12/2016 | Kasese | 0.200002 | 30.13342 |
| 47 | 1/12/2016 | Kasese | 0.333479 | 30.21687 |
| 48 | 1/12/2016 | Kasese | 0.333479 | 30.21687 |
| 49 | 2/12/2016 | Kasese | 0.200002 | 30.13342 |
| 51 | 2/12/2016 | Kasese | 0.233461 | 30.11668 |
| 52 | 2/12/2016 | Kasese | 0.200002 | 30.13342 |
| 53 | 2/12/2016 | Kasese | 0.200002 | 30.13342 |
| 54 | 2/12/2016 | Kasese | 0.233461 | 30.11668 |
| 58 | 2/12/2016 | Kasese | 0.233461 | 30.11668 |
| 63 | 2/12/2016 | Kasese | 0.233461 | 30.11668 |
| 64 | 2/12/2016 | Kasese | 0.233461 | 30.11668 |
| 12A | 19/7/2016 | Kasese | 0.209601 | 30.12505 |
| 13A | 19/7/2016 | Kasese | 0.209601 | 30.12505 |
| 14A | 19/7/2016 | Kasese | 0.209601 | 30.12505 |
| 15A | 19/7/2016 | Kasese | 0.209601 | 30.12505 |
| 18A | 11/11/2016 | Kasese | 0.209601 | 30.12505 |
| 61B | 2/12/2016 | Kasese | 0.233461 | 30.11668 |
| 4 | 28/11/2016 | Kibale | 1.116674 | 31.25005 |
| 9 | 28/11/2016 | Kibale | 1.116674 | 31.25005 |
| 68 | 26/11/2016 | Kiryandongo | 1.666866 | 32.01688 |
| 73 | 26/11/2016 | Kiryandongo | 2.083519 | 32.18358 |
| 79 | 26/11/2016 | Kiryandongo | 1.666866 | 32.01688 |
| 81 | 26/11/2016 | Kiryandongo | 1.76693 | 31.85015 |
| 82 | 26/11/2016 | Kiryandongo | 1.58345 | 32.06668 |
| 89 | 26/11/2016 | Kiryandongo | 1.58345 | 32.06668 |
| 94 | 26/11/2016 | Kiryandongo | 2.200196 | 32.23341 |
| 91A | 21/11/2016 | Kiryandongo | 2.083519 | 32.18358 |
| 10A | 10/11/2016 | Wakiso | 0.51688 | 32.6167 |
| 11A | 19/7/2016 | Wakiso | 0.209601 | 30.12505 |
| 1A | 10/11/2016 | Wakiso | 0.51688 | 32.6002 |
| 3A | 10/11/2016 | Wakiso | 0.51688 | 32.6002 |
| 4A | 10/11/2016 | Wakiso | 0.51688 | 32.6002 |
| 5A | 10/11/2016 | Wakiso | 0.51688 | 32.6002 |
| 6A | 10/11/2016 | Wakiso | 0.51688 | 32.6167 |
| 7A | 10/11/2016 | Wakiso | 0.51688 | 32.6167 |
| 9A | 10/11/2016 | Wakiso | 0.51688 | 32.6167 |

**Footnote:** All samples were collected as larvae except individuals 11A, 12A, 14A, 15A, and 18A which were collected as adult moths. Western districts are: Buliisa, Kabarole, Kamwenge, Kasese, Kibale, Kiryandongo and central district is Wakiso.
